# Maternal health behavior during pregnancy mediates the relationship between maternal stress and newborn telomere length

**DOI:** 10.1016/j.bbih.2026.101269

**Published:** 2026-05-28

**Authors:** Karin de Punder, Malvika Godara, Niklas Speckle, Dries S. Martens, Heiko Klawitter, Nora K. Moog, Claudia Lazarides, Saphira G. La Nave, Wolfgang Henrich, Christine Heim, Thorsten Braun, Karen Lindsay, Pathik D. Wadhwa, Claudia Buss, Sonja Entringer

**Affiliations:** a Charité Universitätsmedizin Berlin, Corporate Member of Freie Universität Berlin and Humboldt-Universität zu Berlin, Institute of Medical Psychology, Berlin, Germany; b Department of Psychology, Clinical Psychology-II, University of Innsbruck, Innsbruck, Austria; c Environmental and Molecular Epidemiology Group, Centre for Environmental Sciences, Hasselt University, Diepenbeek, Belgium; d Institute of Medical Psychology, Heidelberg University Hospital, Heidelberg, Germany; e Department of Psychology, Division Evaluation, University of Freiburg, Freiburg im Breisgau, Germany; f INSERM, UMR1153, Epidemiology and Biostatistics Sorbonne Paris Cité Villemin, Paris, France; g Charité Universitätsmedizin Berlin, Corporate Member of Freie Universität Berlin, Humboldt-Universität zu Berlin, Department of Obstetrics and Experimental Obstetrics, Berlin, Germany; h Center for Safe and Healthy Children, College of Health and Human Development, Pennsylvania State University, Pennsylvania, USA; i The German Centre for Mental Health, Germany; j Susan Samueli Integrative Health Institute, College of Health Sciences, University of California, Irvine, CA, USA; k Department of Pediatrics, School of Medicine, University of California, Irvine, CA, USA; l Development, Health and Disease Research Program, School of Medicine, University of California, Irvine, CA, USA

**Keywords:** Health behaviors, Maternal stress, Newborn, Pregnancy, Telomere length

## Abstract

**Background:**

Health behaviors play a key role in age-related disease risk and are influenced by psychosocial stress. The period of embryonic and fetal life represents among the most sensitive developmental windows at which the effects of maternal behaviors can impact the initial (newborn) setting of telomere length (TL), a marker of biological aging. In the present study, we aimed to investigate the aggregated effect of maternal diet, physical activity and sleep during pregnancy on newborn TL, as well as the mediating role of maternal health behaviors in the relationship between perceived stress and newborn TL.

**Methods:**

Diet quality (Mediterranean Diet Score), physical activity (Measurement of Physical Activity and Sport Questionnaire) and sleep quality (Pittsburgh Sleep Quality Index) were assessed in healthy pregnant women enrolled in a longitudinal prospective cohort study and a Behavioral Health Score (BHS) was computed as the sum of their z-scores. Perceived stress was assessed using the Perceived Stress Scale. TL was measured in cord blood mononuclear cells (CBMCs) collected at birth in 113 newborns.

**Results:**

A higher BHS during pregnancy was associated with longer CBMC newborn TL (*β* = 0.24, 95% CI: 0.006 to 0.05, *p* = .01). The relationship between maternal perceived stress and newborn CBMC TL was mediated by the BHS (*b* = −0.01, bootstrap 95% CI: −0.03 to −0.004).

**Conclusion:**

Promoting maternal stress management and positive health behavior during pregnancy may have lasting benefits for offspring biological aging and health, potentially reducing the burden of age-related diseases across the lifespan.

## Highlights

• Health behaviors such as diet, physical activity and sleep influence aging processes.
• Healthier behavior during pregnancy was linked to longer newborn telomere length.
• Maternal stress affected newborn telomere length through health behaviors.

## 1 Introduction

Health behaviors such as diet, physical activity and sleep significantly influence the aging process (Peel et al., 2005). Telomere length (TL) shortening is considered a hallmark of biological aging and has been associated with risk of a wide range of common physical and mental disorders and mortality (de Punder and Karabatsiakis, 2024; Entringer et al., 2018). On the other hand, positive health behaviors have been linked to longer telomeres and a slower rate of telomere attrition (de Punder and Karabatsiakis, 2024). The telomere biology system is a highly conserved evolutionary mechanism that is essential for preserving genomic and cellular integrity. It encompasses both the structure and function of two key components: telomeres and the reverse transcriptase enzyme telomerase. Telomeres consist of repetitive DNA sequences and associated shelterin protein complexes that protect the ends of linear chromosomes. With each round of cell division, telomeres progressively shorten as a consequence of the 3′ end replication problem (Blackburn, 2005). When telomeres shorten to a critical length, cells typically enter senescence, which serves a protective role by removing or silencing potentially harmful cells but can also induce damage at the organismal level. To compensate for cellular turnover and loss of telomeres due to replication, the level of telomerase activity can be upregulated in cells that undergo rapid expansion, including stem cells and activated immune cells. However, although, these cells can induce telomerase, their telomeres do shorten with age, as indicated by animal and human studies showing telomere shortening in parallel with the accumulation of markers of cell senescence (de Punder et al., 2019; de Punder and Karabatsiakis, 2024).

A critically important characteristic of an individual's telomere biology system is the initial (newborn) setting of TL, which together with telomere attrition rate, is considered a major determinant of TL measured at any subsequent age (Entringer et al., 2018; Martens et al., 2021). This is further supported by evidence indicating that during early life there is a faster attrition rate of telomeres compared to attrition rates seen in adults, likely corresponding to the fast expansion of stem cell pools at the beginning of life (Sidorov et al., 2009). A reduction in newborn TL could therefore confer greater susceptibility in later life for pathophysiological aging-related outcomes (Martens et al., 2022), highlighting the importance of identifying factors that determine an individual's initial setting of TL.

The determinants of inter-individual variation in newborn TL are still not fully understood. While heritability estimates are fairly high, genetic variants associated with TL account for only a small proportion of the observed variance in TL (Aviv, 2012; Prescott et al., 2011). Previous work, including our own, showed that psychosocial stress during pregnancy is linked with shorter newborn (Entringer et al., 2013; Moshfeghinia et al., 2023; Verner et al., 2021) and adult TL (Entringer et al., 2011).

Health-related behaviors, including diet, physical activity and sleep quality comprise modifiable health factors that can influence pregnancy outcomes and the health of the fetus (Weerth, 2018). For example, suboptimal maternal diet and nutritional status during pregnancy are associated with unfavorable pregnancy outcomes, including preterm birth, low infant birth weight (Chia et al., 2019; Gete et al., 2020; Martin et al., 2015) and preeclampsia (Perry et al., 2022). Further, a growing body of evidence shows that physical activity during pregnancy reduces the risk of developing gestational diabetes mellitus, preeclampsia, and hypertensive disorders (Surita et al., 2020) and can prevent possible metabolic disturbances in the offspring (Moreno-Fernandez et al., 2020). Finally, studies have shown that poor sleep quality can predict the duration and type of delivery, reduce the quality of life and increase the risk of gestational hypertension, gestational diabetes mellitus and can affect neonatal outcomes such as birth weight and Apgar scores (Lu et al., 2021;Palagini et al., 2014; Surita et al., 2020).

Moreover, numerous cross-sectional and longitudinal studies have demonstrated associations of such health behaviors and the telomere system in human adults (Canudas et al., 2020; Carroll et al., 2016; Grieshober et al., 2019; Jackowska et al., 2012; Krauss et al., 2011; Tempaku et al., 2015). So far, longitudinal studies on the effects of maternal health behaviors during pregnancy on newborn TL are mostly lacking. Few studies reported positive associations of the maternal dietary intake or blood levels of several vitamins (vitamin C, D and folate) (Entringer et al., 2015a; Kim et al., 2018; Myers et al., 2021) and omega-3 fatty acids with newborn cord blood TL (Liu et al., 2022). The effect of different aspects of maternal sleep on newborn TL have been investigated in three papers with varying results (Ämmälä et al., 2020; Liu et al., 2023; Salihu et al., 2015).

Many health-related behaviors typically do not occur as separate behaviors, but often coexist (Mudryj et al., 2019). For example, physical activity and sleep habits have an impact on dietary patterns and vice versa (Godos et al., 2021; Joo et al., 2019), and a substantial amount of research has shown that getting regular exercise can improve sleep (Dolezal et al., 2017). Given the interrelatedness of these behaviors, we elected to operationalize maternal health behavior using an aggregated score, termed the Behavioral Health Score (BHS), based on measurements of maternal diet quality, physical activity and sleep quality during pregnancy.

Psychosocial stress represents a significant factor with the potential to influence both maternal health behaviors and newborn telomere biology (Entringer et al., 2018; Lindsay et al., 2017; Moshfeghinia et al., 2023). Evidence indicates that psychosocial stress during pregnancy adversely affects key maternal health behaviors, including dietary intake, physical activity, and sleep quality, all of which are critical for favorable pregnancy outcomes (Weerth, 2018). The interconnection between psychosocial stress, maternal health behaviors, and telomere biology is particularly salient during pregnancy, as maternal stress may exert an indirect influence on newborn TL via its impact on maternal health behaviors, underscoring the importance of investigating this potential mechanistic pathway.

Therefore, in the present study, we hypothesized that healthier maternal behavior (i.e., as reflected in a higher BHS) during pregnancy is associated with longer TL measured in cord blood mononuclear cells (CBMC). Furthermore, it was hypothesized that maternal health behavior mediates the relationship between psychosocial stress and newborn TL.

## 2 Materials and methods

### 2.1 Participants

Healthy pregnant women were recruited to participate in a prospective, longitudinal cohort study conducted at the Institute of Medical Psychology and the Department of Obstetrics at the Charité Universtitätsmedizin Berlin, Germany. The study included healthy adult women with singleton, intrauterine pregnancies who intented to deliver at the university hospital. Maternal exclusion criteria were placental or umbilical cord anomalies, major medical comorbidities (e.g., hypertension, diabetes), antenatal systemic corticosteroid exposure, use of psychotropic medications, and illicit drug use. Newborn exclusion criteria were congenital malformations, chromosomal abnormalities, major neonatal complications, and preterm birth before 34 weeks’ gestation. All study procedures were approved by the ethical board of Charité Universitätsmedizin Berlin, and all participants provided written informed consent.

### 2.2 Procedures

The study employed a prospective, longitudinal design with serial assessments over the course of gestation. Women were recruited prior to 16 weeks gestation (T1) followed with an assessment in late pregnancy (T2, 30-34 weeks gestation). Study visit procedures included administration of structured sociodemographic and psychosocial interviews and questionnaires and abstraction of medical and previous obstetric history. At birth (T3) a cord blood sample was obtained by medical staff.

### 2.3 Mediterranean Diet Score

Dietary intake was assessed once in late pregnancy (30-34 weeks gestation, T2) using a validated food frequency questionnaire (Nagl et al., 2016). Women were asked about the intake (frequency and amount) of a variety of food products and beverages over the last month. The Mediterranean diet is a well-researched dietary pattern that has been associated with numerous health benefits, including improved pregnancy and aging-related outcomes (Canudas et al., 2020; Chatzi et al., 2012; Chia et al., 2019; Gete et al., 2020). Therefore, the Mediterranean Diet Score (MDS) was computed from the food frequency questionnaire according to the intake of nine dietary components (fruits, vegetables, legumes, wholegrain products, fish, dairy products, red and processed meat, nuts and olive oil) based on a previously published and validated MDS questionnaire that was adapted for pregnancy (Chatzi et al., 2017; Lindsay et al., 2020).

### 2.4 Physical activity

Physical activity was assessed once in late pregnancy (30-34 weeks gestation, T2) using the exercise and sports activity questionnaire (BSA, Fuchs et al., 2015). The BSA is a validated German questionnaire that measures self-reported movement activity, exercise, and sport behavior over the last month. Total physical activity was calculated in min/week and was naturally log-transformed to account for deviances from normal distribution.

### 2.5 Sleep quality

The Pittsburgh Sleep Quality Index (PSQI, Buysse et al., 1989) was used to assess sleep quality over the previous month in early (T1) and late (T2) pregnancy. There are 19 items which were summed up to compute the PSQI global score that ranges between 0 and 21, where higher scores reflect worse sleep quality. Because the PSQI scores at T1 and T2 were highly correlated (*r* = 0.64, *p* < .001), the mean value of the two time points after z-scoring (to standardize values to the gestation-specific mean) was used for computing the BHS.

### 2.6 Computation of the Behavioral Health Score (BHS)

We operationalized maternal health behavior as an aggregated Behavioral Health Score (BHS) because diet, physical activity, and sleep influence one another during pregnancy, forming an interrelated pattern rather than isolated behaviors. Composite lifestyle indices often outperform single behaviors in predicting health outcomes (Hu et al., 2022). Moreover, aggregation also reduces collinearity and limits multiple testing. The BHS was computed as the sum of z-scores of the MDS, the total amount of physical activity and the mean PSQI global score (after inversion), according to the following formula: BHS = *z*(MDS) + *z*(Ln(physical activity_min/wk_)) - mean{*z*(PSQI_T1_), *z*(PSQI_T2_)}. Higher scores in the BHS reflect more favorable health behaviors. The correlations among the BHS and its individual components are shown in Table S1.

### 2.7 Perceived stress

Perceived (chronic) psychological stress over the past month was quantified using the 10-item version of the Perceived Stress Scale (PSS) (Cohen et al., 1983) in early (T1) and late (T2) pregnancy. The PSS scores at T1 and T2 were moderately to highly correlated (*r* = 0.51, *p* < .001), so the mean value of the two time points (after z-scoring) was used in the mediation analysis.

### 2.8 Psychiatric symptoms

At both pregnancy time points (T1, T2), the Center for Epidemiologic Studies Depression Scale (CES-D) was used to assess depression symptoms (Hautzinger, 1988; Radloff, 1977) and state anxiety was measured using the State subscale of the State-Trait Anxiety Inventory (STAI-S, Laux et al., 1981). CES-D and STAI-S scores were highly correlated with PSS scores at both time points (correlation coefficients ranging from 0.58 to 0.77), and were therefore not included as predictors or covariates in the current analysis to avoid the issue of collinearity. For descriptive purposes, CES-D and STAI-S scores at T1 and T2 are reported in Table 1.

**Table 1:** Maternal sociodemographic and obstetric, behavioral, and psychological characteristics of the study sample.

Maternal characteristics, *n* = 113	Mean (SD), frequency (%)
Maternal age (at birth), years, M (SD)	32.91 (4.11)
SES index, M (SD)	12.41 (1.76)
Country of birth	
Germany, n (%)	95 (84.1)
Pre-pregnancy BMI, M (SD)	23.03 (4.73)
Parity	
Nulliparous, n (%)	69 (61.1)
Multiparous, n (%)	44 (38.9)
Gestational age at birth, weeks, M (SD)	39.35 (1.15)
Child sex, female, n (%)	52 (46.0)
MDS, M (SD)	2.91 (1.55)
Physical activity (min/week), M (SD)	956.02 (951.69)
PSQI global score, T1, M (SD)	5.65 (2.56)
PSQI global score, T2, M (SD)	6.2 (3.01)
PSS, T1, M (SD)	14.49 (6.69)
PSS, T2, M (SD)	12.02 (7.06)
CES-D, T1, M (SD)	12.56 (7.91)
CES-D, T2, M (SD)	11.50 (7.96)
STAI-S, T1, M (SD)	36.58 (8.51)
STAI-S, T2, M (SD)	35.15 (9.70)

Abbreviations: BMI, Body mass index; CES-D, Center for Epidemiologic Studies Depression Scale; MDS, Mediterranean diet score; PSS, Perceived Stress Scale; PSQI, Pittsburgh Sleep Quality Index; SES, Socioeconomic status; STAI-S, State–Trait Anxiety Inventory (State subscale).

### 2.9 CBMC isolation

Cord blood was collected by medical staff immediately after birth using BD Vacutainer CPT (BD Vacutainer, Cat. 362760). Samples were centrifuged at room temperature for 20 min at 1600 x g and transported within 24 h to the Laboratory of the Institute of Medical Psychology, Charité Universtitätsmedizin Berlin, Germany, for further processing. Cord blood mononuclear cells (CBMCs) were isolated according to the manufactures’ protocol for the BD Vacutainer CPT system and counted using a hemocytometer. A total of 1 x 10^7^ cells, were dissolved in 1 ml of freezing media consisting of 10% DMSO (Sigma-Aldrich, D2438-5X10ML) in FBS (HyClone, SH3007102). Cells were frozen using a Mister Frosty at −80 °C and after 24h transferred to liquid nitrogen for long term storage.

### 2.10 Measurement of TL

Newborn average relative TL was measured in CBMCs at the Laboratory of the Centre for Environmental Sciences, University Hasselt, Belgium, using a modified singleplex quantitative PCR (qPCR) method adapted from Cawthon (2002) and Cawthon (2009). DNA was extracted in one batch from CBMC using the QIAamp DNA Mini Kit (Qiagen, Inc., Venlo, The Netherlands). To ensure a uniform DNA input of 5 ng for each qPCR reaction, samples were diluted and checked using the Qubit™ dsDNA High Sensitivity Assay Kit (Life Technologies, Europe) using the Qubit™ Flex Fluorometer (Life Technologies, Europe). All samples were measured in triplicates on a QuantStudio 5 real-time PCR system (Applied Biosystems) in a 384-well format. First, a single copy gene (human β globin) reaction was performed containing 5 ng DNA template, 1x KAPA SYBR® FAST, Low ROXTM master mix (Kapa Biosystems, Merck), 450 nM HBG1 primer (GCTTCTGACACAACTGTGTTCACTAGC), and 450 nM HBG2 primer (CACCAACTTCATCCACGTTCACC). Cycling conditions were as follows: 1 cycle at 95 °C for 3 min, 40 cycles at 95 °C for 3 s, and 58 °C for 15 s. Second, a telomere-specific reaction was performed, containing 5 ng DNA template, 1x KAPA SYBR® FAST, Low ROXTM master mix (Kapa Biosystems, Merck), 2 mM DTT, 100 nM TelG primer (ACACTAAGGTTTGGGTTTGGGTTTGGGTTTGGGTTAGTGT), and 100 nM TelC primer (TGTTAGGTATCCCTATCCCTATCCCTATCCCTATCCCTAACA). Cycling conditions were as follows: 1 cycle at 95 °C for 3 min, 2 cycles at 94 °C for 3 s and 49 °C for 15 s, and 30 cycles at 94 °C for 3 s, 62 °C for 5 s, and 74 °C for 10 s. On each run, PCR efficiency was evaluated using a standard 6-point serial diluted DNA standard curve (efficiency was 101% for Tel, and 98% for HBG with an R2 > 0.99). The final average relative TL was calculated as a normalized relative quantity (NRQ) using the qBase software (Biogazelle, Zwijnaarde, Belgium). First, the relative quantity (RQ) is calculated based on the delta-Cq method for telomere (T) and single-copy gene (S) obtained Cq values. As the choice of a calibrator sample (sample to which subsequent normalization is performed, delta-delta-Cq) strongly influence the error on the final RQs (as a result of the measurement error on the calibrator sample), normalization is performed to the arithmetic mean quantification values for all analyzed samples, which results in the NRQ. Mathematical calculation formulas to obtain RQ, and NRQ are provided by Hellemans et al. (2007). CVs within triplicates of the telomere runs, single-copy gene runs, and T/S ratios were achieved of 0.52 %, 0.26 %, and 5.47 %, respectively. In addition, the average measure intra-class correlation coefficient of the T/S ratio measurements was .93 with a 95% confidence interval (CI) from 0.91 to 0.94, *p* < .0001.

### 2.11 Covariates

Covariates were specified a priori based on previously established associations with newborn TL and/or behavioral factors and included maternal age (Jardí et al., 2019; Okuda et al., 2002), socioeconomic status (SES) (Martens et al., 2020), pre-pregnancy BMI (Martens et al., 2016), parity (the number of times a female has given birth) (Houminer-Klepar et al., 2023; Rockliffe et al., 2021), child sex (Lansdorp, 2022) and gestational age at birth (Vasu et al., 2017). Given that smoking is an important behavioral factor that could influence newborn TL (Ip et al., 2017; X. Liu et al., 2024; Rumrich et al., 2020; Theall et al., 2013), but was relatively uncommon in our sample (12/125 women), we excluded participants who reported smoking during early and late pregnancy from the analyses. SES was calculated using an adapted version of the method by Lampert et al. (2013) and was based on maternal educational level (originally assessed in categories from less than high school to advanced degree (master/doctorate) and then recoded into values from 1 to 7) and household income (recoded into values from 1 to 7). Net equivalent incomes were calculated and adjusted for inflation (Ha et al., 2023) in accordance with the OECD equivalence scale. Because there was minimal variability in ethnicity/country of origin in our sample, this variable was summarized descriptively (see Table 1) and not included as a covariate.

### 2.12 Statistics

Descriptive statistics, correlation and regression analyses were conducted using IBM SPSS Statistics (SPSS 29.0, Inc., Chicago, IL, USA). Two-tailed Pearson's correlation analyses were conducted to assess the direction and magnitude of the relationships between all variables included in the analyses, as presented in Table S2 of the supplementary materials. Regression analyses were applied to test the relationship between the maternal BHS and newborn TL, with and without the inclusion of the covariates maternal age, SES, pre-pregnancy BMI, parity, child sex and gestational age at birth. Next, the PROCESS (Hayes, 2022) macro model 4 was used to test the mediating role of the BHS in the relationship between perceived stress and newborn TL. In a second step, the a priori selected covariates were included. We conducted ordinary least squares (OLS) path analyses using 5000 bootstrapping samples and a 95% confidence interval (CI). For all analyses, *p* < .05 was considered significant.

## 3 Results

### 3.1 Participant's characteristics

A total of 475 pregnant women were enrolled, of whom complete data on maternal stress and health behaviors and newborn TL were available for n = 113 participants. Reductions from the enrolled cohort include lack of infant biospecimen collection and incomplete stress and health behavior assessments. Compared with the full cohort, the mother-child dyads with complete data differed only in SES, *t* (400) = −4.63, *p* < 0.001, such that participants included in the analyses had a higher SES (M = 12.41, SD = 1.76) than the total group (M = 11.19, SD = 2.59). All newborns included in the analyses were born full-term (≥37 weeks’ gestational age) and had no known congenital, genetic, or neurological disorders. The characteristics of the study participants are presented in Table 1.

### 3.2 Relationship between the BHS and newborn TL

**Fig. 1:**
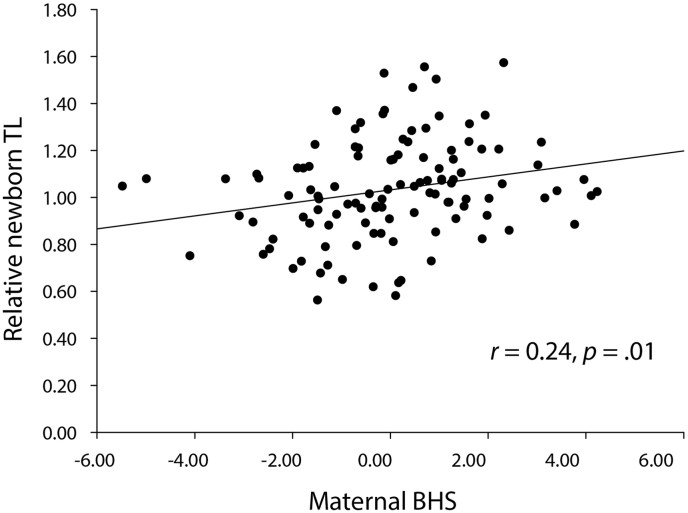
Association of the maternal Behavioral Health Score (BHS) and newborn telomere length (*r* = 0.24, *p* = .01, *n* = 113). TL, relative T/S ratio.

We first examined the relationship between the BHS and newborn TL. In unadjusted analysis, the BHS was significantly and positively associated with newborn TL (*b* = 0.03, *β* = 0.24, 95% CI: 0.006 to 0.05, *p* = .01, Fig. 1), indicating that each SD increase of the BHS, results in a 0.24 SD increase in newborn TL. After inclusion of the a priori selected covariates maternal age, SES, pre-pregnancy BMI, parity, child sex and gestational age at birth in the regression model, the association between the BHS and TL remained significant (*b* = 0.03, *β* = 0.21, 95% CI: 0.002 to 0.05, *p* = .03). None of the covariates showed a significant association with newborn TL (all *p*-values >0.05). For a detailed overview of the regression statistics, please refer to Table S3. Results from additional regression analysis assessing the associations between individual components of the BHS and newborn TL are shown in Table S4.

### 3.3 Mediation model

**Fig. 2:**
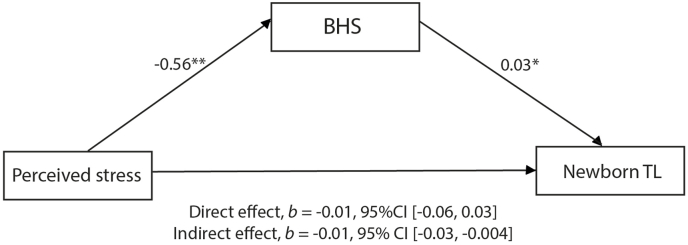
Mediation model illustrating the role of the Behavioral Health Score (BHS) as a mediator between perceived stress (PSS, mean value across pregnancy, exposure) and newborn telomere length (TL, outcome). Arrows show unstandardized path coefficients (*b*): PSS → BHS, *b* = −0.56; BHS → TL, *b* = 0.03. The horizontal arrow depicts the (non-significant) direct effect of PSS on TL, *b* = −0.01, CI 95%: −0.06 to 0.03. The indirect effect via BHS (product of the two diagonal paths) was significant, *b* = −0.01 95% CI: −0.03 to −0.004 (the confidence interval does not include zero). ∗*p* < .05; ∗∗*p* < .01.

Next, the mediating role of the BHS in the relationship between perceived stress and newborn TL was investigated. Fig. 2 presents the model with the path coefficients shown on the arrows. In the model, perceived stress was significantly and negatively associated with the BHS (*b* = −0.56, CI 95%: −0.94 to −0.18), and the BHS was significantly positively related to newborn TL (*b* = 0.03, CI 95%: 0.004 to 0.05). Higher perceived stress levels were not directly associated with shorter newborn TL (*b* = −0.01, CI 95%: −0.06 to 0.03). However, there was a significant indirect effect of perceived stress on newborn TL through the BHS (*b* = −0.01, bootstrap 95% CI: −0.03 to −0.004), indicating that perceived stress primarily influenced newborn TL indirectly by affecting maternal health behavior. After inclusion of the covariates maternal age, SES, pre-pregnancy BMI, parity, child sex and gestational age at birth, the indirect effect remained significant (*b* = −0.01, bootstrap 95%: −0.03 to −0.001).

## 4 Discussion

The present study set out to examine the aggregate effect of maternal health behavior (diet, physical activity and sleep) during pregnancy on newborn TL and to assess its mediating role in the relationship between psychosocial stress and newborn TL. In line with our hypothesis we found that more positive health behavior during pregnancy was associated with longer newborn CBMC TL. To our knowledge, this is the first study to demonstrate that an aggregate index of prenatal health behaviors is associated with CBMC newborn TL, moving beyond prior work focused on single factors or (micro)nutrients. Further, we showed that perceived stress was indirectly associated to newborn TL through its negative effect on maternal health behavior. Taken together these findings indicate that prenatal stress may impact newborn telomere biology via potentially modifiable behavioral factors, with consequences for long-term health and lifespan.

Until now, not many studies investigated the longitudinal effects of maternal health behaviors on offspring telomere biology. So far, the Mediterranean diet and physical activity have only been associated with longer leukocyte TL in non-pregnant adults (Canudas et al., 2020; Krauss et al., 2011; Puterman et al., 2010, 2018). However, several studies have demonstrated associations between maternal dietary intake or blood concentrations of specific vitamins (vitamin C, D and folate) (Entringer et al., 2015a; Kim et al., 2018; Myers et al., 2021) and omega-3 fatty acids (Liu et al., 2022), which typically reflect adherence to a Mediterranean dietary pattern, with newborn cord blood TL. The impact of maternal sleep behaviors on newborn TL has been investigated in three papers (Ämmälä et al., 2020; Liu et al., 2023; Salihu et al., 2015). A longitudinal prospective study found no association between maternal sleep quality and newborn TL despite a large sample size of 1405 mother-newborn dyads (Ämmälä et al., 2020). However, sleep quality in that study was assessed only in late pregnancy and evaluated with a different instrument from which an insomnia score was calculated (Partinen and Gislason, 1995). Similar to our findings, Liu et al. (2023) showed that poor maternal sleep quality (assessed by interview) was associated with shorter cord blood TL. Also, maternal symptoms of sleep disordered breathing were found to be related to shorter TL (Salihu et al., 2015). Overall, the observed associations between maternal health behaviors and offspring telomere biology support the hypothesis that modifiable prenatal behaviors may influence the newborn TL set point.

Contrary to the finding of a recent meta-analysis (Moshfeghinia et al., 2023), which reported a significant relationship between maternal psychological stress and newborn TL across eight studies, perceived stress during pregnancy in our study was not associated with newborn TL. This could be a power issue due to the relatively small sample size. However, the mediation model revealed that perceived stress indirectly influenced newborn TL through its impact on maternal health behaviors. This observation is in line with the model suggested by Weerth et al. (Weerth, 2018), who proposed that maternal health behaviors constitute essential mediators between maternal prenatal stress and fetal programming processes. Moreover, this finding suggests that interventions targeting stress reduction during pregnancy may improve maternal health behaviors and, in turn, positively influence offspring telomere biology.

Adherence to the Mediterranean diet, regular physical activity and good sleep quality have been shown to exert antioxidant and anti-inflammatory effects, as well as positively modulating endocrine function and metabolism in both pregnant and non-pregnant populations (Bublitz et al., 2018; Davinelli et al., 2019; de Punder and Karabatsiakis, 2024; Weerth, 2018). Therefore, the effect of positive prenatal health behaviors on newborn telomere biology may be mediated by the programming actions of maternal–placental–fetal oxidative, immune, endocrine and metabolic pathways (Entringer et al., 2018).

Telomeres are guanine-rich and thus especially vulnerable to oxidative lesions. As a result, oxidative stress markedly accelerates telomere attrition, and triggers cellular senescence or apoptosis via DNA damage-mediated activation of the p53 pathway (von Zglinicki, 2002). Hypertensive pregnancy disorders have been linked with shorter trophoblast TL (Biron-Shental et al., 2015) and increased oxidative stress levels in both mothers and newborns (Bharadwaj et al., 2018). Interestingly, higher maternal antioxidant intake mitigated the negative influence of metals on newborn leukocyte TL (Cowell et al., 2020).

Inflammatory mediators, including C-reactive protein (CRP), interleukin-6 (IL-6), and tumor necrosis factor-α (TNF-α), have been associated with telomere shortening (de Punder and Karabatsiakis, 2024; Lazarides et al., 2019). Immune activation accelerates telomere erosion by increasing proliferation rates and oxidative stress levels (de Punder and Karabatsiakis, 2024). A pro-inflammatory state during pregnancy was associated with shorter offspring immune cell TL (Lazarides et al., 2019), indicating that promoting anti-inflammatory behavioral pathways during pregnancy my positively impact the initial setting of newborn telomere biology.

Neuroendocrine stress hormones, including cortisol and DHEA, have been associated in human studies with shorter and longer telomeres, respectively (de Punder et al., 2023, 2024; Jiang et al., 2019). Also, it has been reported that immune cells from individuals with greater cortisol stress reactivity showed a reduced capacity to upregulate telomerase activity, which in turn, was associated with shorter TL (de Punder et al., 2018, 2023). Regular physical activity stimulates circulating DHEA levels (Keller et al., 2021), providing a behavioral pathway that may support longer TL. Consistent with this, pregnancy-related research has reported a positive association between cord blood DHEA and newborn TL (Liu et al., 2017). Finally, other studies have reported associations between maternal estriol levels (Entringer et al., 2015b) and vitamin D status (Kim et al., 2018) during pregnancy with newborn TL.

In humans, adverse metabolic profiles, including higher adiposity, insulin resistance, hyperglycemia (e.g., elevated fasting glucose and HbA1c), and dyslipidemia (high triglycerides, low HDL cholesterol), are consistently associated with shorter leukocyte TL (Cheng et al., 2021). Chronic hyperglycemia and advanced glycation end-products increase reactive oxygen species, DNA damage and inflammation (Kavitha et al., 2025), and excess circulating fatty acids promote lipotoxicity and pro-inflammatory adipokine signaling (Gugliucci, 2022). In human pregnancy studies, gestational diabetes mellitus was associated with shorter leukocyte TL (Liu et al., 2024; Xu et al., 2014), and negative correlations between maternal and fetal glucose and leukocyte TL and telomerase activity have been observed (Gilfillan et al., 2016; S. Liu et al., 2024).

Thus, modifiable maternal health behaviors and stress-reduction pathways, may help create a more favorable endocrine, and metabolic milieu and reduce oxidative stress and inflammation during pregnancy, thereby supporting healthier initial telomere biology in the newborn.

Key strengths of the present study are its prospective design and the comprehensive examination of maternal stress and health behaviors during pregnancy. Additionally, because we measured newborn TL immediately after birth, these measures are unlikely to have been confounded by postnatal factors.

The study is also limited in several ways. First, newborn TL was not adjusted for CBMC subsets. However, it appears that within-individual TL across lymphocyte subpopulations is correlated, whereas substantial variation between subjects is observed (Lin et al., 2010). Moreover, studies with humans umbilical cord blood and other newborn tissues showed strong correlations of TL among leukocyte subsets and hematopoietic progenitor cells (Kimura et al., 2010), and among leukocytes and umbilical artery and foreskin tissue (Okuda et al., 2002). These findings indicate considerable synchrony of newborn TL across different human tissues and cell types and given that hematopoietic stem cells ensure the production of blood cells through self-renewal and differentiation into all blood lineages, newborn CBMC TL may represent a good indicator of general immune cell telomere dynamics at birth (Kimura et al., 2010). Second, the sample size was relatively small, potentially increasing the risk of type II errors. Third, our sample consisted primarily of healthy, non-smoking mothers with relatively high SES, limiting the general validity of our findings and highlighting the importance of replication in larger clinical cohorts. Fourth, all behavioral measures relied on self-reports, and both maternal diet quality and physical activity were assessed only during late pregnancy, which limited the ability to capture behavioral patterns across the entire pregnancy. Fifth, we did not consider maternal psychiatric symptomology, which may represent a relevant source of residual confounding, given the well-established associations between maternal mental health, health behaviors, and offspring biological outcomes, including TL (Stout-Oswald et al., 2022). Finally, our study only assessed child TL at birth. In future research, the trajectories of telomere attrition should be tracked over a longer period after birth to cover different periods of human development.

To conclude, we observed a positive prospective association of maternal health behaviors with newborn TL and that maternal perceived stress during pregnancy indirectly influences newborn TL through its impact on health behaviors. Since newborn TL is an important determinant of telomere biology-related processes (Martens et al., 2021), and possible subsequent health and disease outcomes later in life (Martens et al., 2022), our data suggest that improving maternal health behaviors during pregnancy may have long-lasting effects on offspring health and potentially influence future generations. Equitable access to prenatal care that addresses psychological stress while supporting healthy lifestyle behaviors should be a public health priority given the potential implications for reducing the burden of age-related diseases and improving health outcomes across the lifespan.

## CRediT authorship contribution statement

**Karin de Punder:** Formal analysis, Investigation, Visualization, Writing – original draft. **Malvika Godara:** Data curation, Formal analysis. **Niklas Speckle:** Formal analysis, Writing – review & editing. **Dries S. Martens:** Formal analysis, Investigation. **Heiko Klawitter:** Investigation, Writing – review & editing. **Nora K. Moog:** Investigation, Writing – review & editing. **Claudia Lazarides:** Investigation, Writing – review & editing. **Saphira G. La Nave:** Investigation, Writing – review & editing. **Wolfgang Henrich:** Resources, Writing – review & editing. **Christine Heim:** Resources, Writing – review & editing. **Thorsten Braun:** Resources, Writing – review & editing. **Karen Lindsay:** Formal analysis, Writing – review & editing. **Pathik D. Wadhwa:** Conceptualization, Writing – review & editing. **Claudia Buss:** Conceptualization, Funding acquisition, Methodology, Project administration, Supervision, Writing – review & editing. **Sonja Entringer:** Conceptualization, Funding acquisition, Methodology, Project administration, Supervision, Writing – review & editing.

## Funding

This work was supported by ERC-Stg 678073 and ERC-Stg 639766. Dries S. Martens was supported by a postdoctoral grant from the Research Foundations Flanders (FWO grant 12X9623N).

## Declaration of competing interest

The authors declare that they have no known competing financial interests or personal relationships that could have appeared to influence the work reported in this paper.

## Data Availability

Data will be made available on request.
