# Supplementary material for: Maternal health behavior during pregnancy mediates the relationship between maternal stress and newborn telomere length
Source: Brain Behav Immun Health. 2026 May 28;55:101269. doi: 10.1016/j.bbih.2026.101269 (PMC13276604; doi:10.1016/j.bbih.2026.101269)
Supplement: Multimedia component 1 [file mmc1.docx]

**Table S1**. Correlations among the BHS and its individual components.

| **Variables** |  | 1 | 2 | 3 | 4 |
| --- | --- | --- | --- | --- | --- |
| 1. BHS | *r* | - |  |  |  |
|  | *p* | - |  |  |  |
| 2. MDS, T2 | *r* | .66*** | - |  |  |
|  | *p* | <.001 | - |  |  |
| 3. Physical activity (Ln), T2 | *r* | .68*** | .19* | - |  |
|  | *p* | <.001 | .04 | - |  |
| 4. PSQI, mean T1, T2 | *r* | -.53*** | -.02 | -.05 | - |
|  | *p* | <.001 | .86 | .61 | - |

Note, ****p* < .001, **p* < .05. Abbreviations: BHS, Behavioral Health Score; MDS, Mediterranean diet score; PSQI, Pittsburgh Sleep Quality Index.

**Table S2**. Correlation matrix of study variables (*n* = 113).

| **Variables** |  | 1 | 2 | 3 | 4 | 5 | 6 | 7 | 8 | 9 | 10 | 11 | 12 | 13 | 14 |
| --- | --- | --- | --- | --- | --- | --- | --- | --- | --- | --- | --- | --- | --- | --- | --- |
| 1. BHS | *r* | - |  |  |  |  |  |  |  |  |  |  |  |  |  |
|  | *p* | - |  |  |  |  |  |  |  |  |  |  |  |  |  |
| 2. PSS T1 | *r* | -.29** | - |  |  |  |  |  |  |  |  |  |  |  |  |
|  | *p* | .002 | - |  |  |  |  |  |  |  |  |  |  |  |  |
| 3. PSS T2 | *r* | -.17† | .51*** | - |  |  |  |  |  |  |  |  |  |  |  |
|  | *p* | .08 | <.001 | - |  |  |  |  |  |  |  |  |  |  |  |
| 4. MDS | *r* | .66*** | -.06 | .20* | - |  |  |  |  |  |  |  |  |  |  |
|  | *p* | <.001 | .51 | .03 | - |  |  |  |  |  |  |  |  |  |  |
| 5. Physical activity (Ln) | *r* | .68*** | .03 | -.1 | .19* | - |  |  |  |  |  |  |  |  |  |
|  | *p* | <.001 | .79 | .30 | .04 | - |  |  |  |  |  |  |  |  |  |
| 6. PSQI global score T1 | *r* | -.54*** | .55*** | .40*** | -.11 | -.05 | - |  |  |  |  |  |  |  |  |
|  | *p* | <.001 | <.001 | <.001 | .24 | .64 | - |  |  |  |  |  |  |  |  |
| 7. PSQI global score T2 | *r* | -.43*** | .42*** | .43*** | .09 | -.04 | .64*** | - |  |  |  |  |  |  |  |
|  | *p* | <.001 | <.001 | <.001 | .34 | .68 | <.001 | - |  |  |  |  |  |  |  |
| 8. Maternal age | *r* | .21* | -.05 | .006 | .21* | .17^†^ | -.14 | .12 | - |  |  |  |  |  |  |
|  | *p* | .03 | .63 | .95 | .03 | .08 | .15 | .21 | - |  |  |  |  |  |  |
| 9. Maternal SES | *r* | .18† | -.18† | -.11 | .21* | -.06 | -.20* | -.14 | .29** | - |  |  |  |  |  |
|  | *p* | .06 | .05 | .26 | .03 | .53 | .03 | .14 | .002 | - |  |  |  |  |  |
| 10. Pre-pregnancy BMI | *r* | -.19* | -.11 | -.08 | -.19* | -.08 | .06 | .12 | -.12 | -.12 | - |  |  |  |  |
|  | *p* | .04 | .26 | .40 | .049 | .42 | .56 | .21 | .22 | .19 | - |  |  |  |  |
| 11. Parity | *r* | .27* | .15 | .05 | .06 | .47*** | .006 | .06 | .30** | -.13 | -.09 | - |  |  |  |
|  | *p* | .003 | .11 | .59 | .50 | <.001 | .95 | .52 | .001 | .18 | .33 | - |  |  |  |
| 12. Child sex | *r* | .05 | -.06 | .03 | .007 | .02 | .01 | -.15 | -.05 | -.02 | .09 | .09 | - |  |  |
|  | *p* | .57 | .49 | .77 | .94 | .83 | .92 | .11 | .64 | .85 | .33 | .33 | - |  |  |
| 13. Gestational age at birth | *r* | -.04 | -.002 | .007 | -.04 | -.10 | -.07 | -.06 | -.20* | -.08 | .07 | -.19* | .05 | - |  |
|  | *p* | .67 | .98 | .94 | .69 | .30 | .48 | .54 | .03 | .42 | .46 | .047 | .62 | - |  |
| 14. Newborn TL | *r* | .24* | -.17† | -.03 | .26** | .04 | -.24** | -.02 | .12 | .21* | -.14 | -.06 | .05 | .05 | - |
|  | *p* | .01 | .07 | .73 | .006 | .66 | .009 | .86 | .22 | .03 | .14 | .53 | .63 | .62 | - |

Note, ****p* < .001, ***p* < .01, *p < .05, ^†^*p* < .10 (marginal significance). Abbreviations: BHS, Behavioral Health Score; BMI, Body mass index; MDS, Mediterranean diet score; PSS, Perceived Stress Scale; PSQI, Pittsburgh Sleep Quality Index; SES, Socioeconomic status; TL, Telomere length.

**Table S3.** Results of the regression analysis with the BHS and the covariates maternal age, SES, pre-pregnancy BMI, parity, child sex and gestational age at birth as the predictor variables and newborn TL as the outcome variable, *n* = 113.

| **Effect** | **Estimate *b*** | **SE** | ***β*** | ***t*** | **95% CI LL** | **95% CI UL** | ***p*** |
| --- | --- | --- | --- | --- | --- | --- | --- |
| **BHS** | 0.03 | 0.01 | 0.21 | 2.15 | 0.002 | 0.05 | **.03** |
| **Maternal age** | 0.004 | 0.005 | 0.08 | 0.74 | -0.007 | 0.02 | .46 |
| **SES** | 0.02 | 0.01 | 0.13 | 1.26 | -0.009 | 0.04 | .21 |
| **BMI** | -0.004 | 0.004 | -0.10 | -1.00 | -0.01 | 0.004 | .32 |
| **Parity** | -0.05 | 0.04 | -0.13 | -1.22 | -0.13 | 0.03 | .23 |
| **Child sex** | 0.03 | 0.04 | 0.06 | 0.62 | -0.05 | 0.10 | .54 |
| **Gestational age at birth** | 0.01 | 0.02 | 0.06 | 0.65 | -0.02 | 0.05 | .52 |

Abbreviations: BHS, Behavioral Health Score; BMI, Body Mass Index; CI, confidence interval; LL, lower limit; UL, upper limit;

SES, Socioeconomic status; TL, Telomere length.

**Table S4.** Results of the regression analysis with the individual components of the BHS (diet quality, physical activity and sleep quality) as the predictor variables and newborn TL as the outcome variable, with and without adjusting for the covariates maternal age, SES, pre-pregnancy BMI, parity, child sex, and gestational age at birth (*n* = 113).

| **Effect** | **Estimate *b*** | **SE** | ***β*** | ***t*** | **95% CI LL** | **95% CI UL** | ***p*** |
| --- | --- | --- | --- | --- | --- | --- | --- |
| **Diet quality** (MDS, unadjusted model) | 0.04 | 0.01 | 0.26 | 2.79 | 0.01 | 0.06 | **.006** |
| Adjusted model | 0.03 | 0.01 | 0.21 | 2.10 | 0.002 | 0.06 | **.04** |
| **Physical activity** (minutes/week, Ln, unadjusted model) | 0.009 | 0.02 | 0.04 | 0.45 | -0.03 | 0.05 | .66 |
| Adjusted model | 0.02 | 0.02 | 0.09 | 0.80 | -0.03 | 0.07 | .43 |
| **Sleep quality** (PSQI, mean T1, T2, unadjusted model) | -0.04 | 0.02 | -0.15 | -1.56 | -0.08 | 0.009 | .12 |
| Adjusted model | -0.02 | 0.02 | -0.10 | -.98 | -0.07 | 0.02 | .33 |

Abbreviations: CI, confidence interval; LL, lower limit; UL, upper limit; MDS, Mediterranean diet score; PSQI, Pittsburgh Sleep Quality Index; SE, standard error; SES, Socioeconomic status; TL, Telomere length.
